# Distinct mechanisms mediating therapy-induced cellular senescence in prostate cancer

**DOI:** 10.1186/s13578-022-00941-0

**Published:** 2022-12-15

**Authors:** Julia Kallenbach, Golnaz Atri Roozbahani, Mehdi Heidari Horestani, Aria Baniahmad

**Affiliations:** Institute of Human Genetics, Jena University Hospital, Friedrich Schiller University, Am Klinikum 1, 07740 Jena, Germany

**Keywords:** Cancer cell senescence, Androgen receptor, Chemotherapy, Radiotherapy, Prostate cancer, Senescence-associated secretory phenotype, Exosome

## Abstract

**Background:**

Prostate cancer (PCa) is an age-related malignancy in men with a high incidence rate. PCa treatments face many obstacles due to cancer cell resistance and many bypassing mechanisms to escape therapy. According to the intricacy of PCa, many standard therapies are being used depending on PCa stages including radical prostatectomy, radiation therapy, androgen receptor (AR) targeted therapy (androgen deprivation therapy, supraphysiological androgen, and AR antagonists) and chemotherapy. Most of the aforementioned therapies have been implicated to induce cellular senescence. Cellular senescence is defined as a stable cell cycle arrest in the G1 phase and is one of the mechanisms that prevent cancer proliferation.

**Results:**

In this review, we provide and analyze different mechanisms of therapy-induced senescence (TIS) in PCa and their effects on the tumor. Interestingly, it seems that different molecular pathways are used by cancer cells for TIS. Understanding the complexity and underlying mechanisms of cellular senescence is very critical due to its role in tumorigenesis. The most prevalent analyzed pathways in PCa as TIS are the p53/p21^WAF1/CIP1^, the p15^INK4B^/p16^INK4A^/pRb/E2F/Cyclin D, the ROS/ERK, p27^Kip1^/CDK/pRb, and the p27^Kip1^/Skp2/C/EBP β signaling. Despite growth inhibition, senescent cells are highly metabolically active. In addition, their secretome, which is termed senescence-associated secretory phenotype (SASP), affects within the tumor microenvironment neighboring non-tumor and tumor cells and thereby may regulate the growth of tumors. Induction of cancer cell senescence is therefore a double-edged sword that can lead to reduced or enhanced tumor growth.

**Conclusion:**

Thus, dependent on the type of senescence inducer and the specific senescence-induced cellular pathway, it is useful to develop pathway-specific senolytic compounds to specifically targeting senescent cells in order to evict senescent cells and thereby to reduce SASP side effects.

## Introduction

Prostate cancer (PCa) is one the most diagnosed cancer and the second leading cause of cancer-related death in men in Western countries [1]. Localized PCa is treated by surgical resection including radical prostatectomy, external beam or proton radiotherapy and brachytherapy [2]. The growth and progression of localized PCa as well as the early stage of advanced/metastatic PCa depends on androgen, which mediates its effects through the androgen receptor (AR). Hence, PCa that recurred after local therapy and progressed to the advanced stage, is usually treated with androgen deprivation therapy (ADT) [3]. ADT reduces serum testosterone concentrations by inhibiting the testosterone production along the hypothalamus-testis axis [4, 5]. This approach is achieved preferentially by medical castration using LHRH agonists or antagonists such as goserelin, leuoprolide, degarelix or relugolix that reduce the body-own androgen production [6]. Most patients initially respond with reduced tumor burden and prostate-specific antigen (PSA) level [7]. One possible tumor-suppressive mechanism of ADT is the induction of cellular senescence in PCa tumors of patients [8].

However, after a period of time, PCa becomes resistant to the treatment and progresses despite androgen ablation from an androgen-dependent, castration-sensitive stage to castration-resistant PCa (CRPC) but mostly remains dependent on AR.

The development of resistances against ADT relies on adaptive changes and reactivation of the AR-signaling including intratumoral androgen production within the PCa tissue, AR gene amplification, point mutations in AR and constitutively active AR splice variants [9–11]. Further, the activation of other signaling mechanisms including kinases such as Src-AKT and the MAPK-signaling pathways are involved in androgen-refractory proliferation [9, 12, 13]. At this stage of PCa, ADT is commonly combined with other treatments including abiraterone acetate and AR antagonists (antiandrogens) to improve the outcome of the patients. Abiraterone acetate interferes with the adrenal production of testosterone precursors [14]. Several antiandrogens have been approved for the PCa treatment including first-generation antiandrogens such as bicalutamide, flutamide as well as second-generation non-steroidal AR antagonists like enzalutamide, darolutamide, and apalutamide [15–17]. Second-generation AR antagonists have improved efficacy and potency compared to first-generation antagonists and are effective in metastatic CSPC, non-metastatic and metastatic CRPC [18]. Further, clinical trials investigated the effects of the combination of enzalutamide with chemotherapy by docetaxel in men with metastatic CRPC showing reduced PSA levels and improved outcomes compared to enzalutamide alone [19, 20]. Recent studies indicate that several PCa therapies that includes the AR targeted therapies, radiotherapy, and chemotherapy induce senescence in vitro and in vivo. In the following paragraphs we discuss the different mechanisms of senescence induction by PCa therapies and their effects on the tumor.

## Cellular senescence

Cellular senescence is a multifaceted stress response involved in tumor suppression, tissue repair, aging, as well as cancer therapy [21]. Senescent cells are arrested in the G1 phase of the cell cycle. Senescence provides a mechanism to inhibit cancer cell growth but might have beneficial or adverse effects in a tumor [22]. In general, cellular senescence is mediated through exogenous and endogenous stimulants, causing changes in cell morphology as well as gene expression. Importantly, cellular senescence occurs naturally during vertebrate embryogenesis as a normal program and regulates patterning in development [23, 24].

Naturally, senescent cells accumulate with age in all tissues so far analyzed. Senescent cells remain metabolically active and exhibit a special but diverse secretome termed as senescence-associated secretory phenotype (SASP). SASP contains chemokines and chemokines that may influence the tissue microenvironment including tumor microenvironment [25, 26]. In addition, SASP can induce a systemic pro-inflammation to change and induce an inflammatory process also in the microenvironment [27]. Of note, also the ageing prostate contains senescent cells of which the SASP is associated with benign prostate hyperplasia and PCa [28].

There are two basic types of senescence: an accelerated and a chronic or replicative senescence. Extrinsic stressors, chemical and physical agents such as oxidative stress, chemotherapeutics such as doxorubicin treatment, and X-ray irradiation, may induce accelerated cell senescence. Whereas persistent cellular stress, such as extended proliferation with associated DNA replication, reduced telomere length and accumulation of genomic damages, may trigger chronic senescence [29].

Some hallmarks are associated with senescence, including morphological changes such as enlarged, flattened shape and increased granularity, upregulation of senescence-associated beta galactosidase activity (SA β-gal), increased levels of cell cycle inhibitors, epigenetic changes such as senescence-associated heterochromatin foci (SAHF), nuclear envelope alterations, and expression of the SASP [26, 30, 31]. Although senescent cells can no longer divide, they are metabolically active and secrete factors, which mediate paracrine effects on neighboring non-senescent cells in the tissue microenvironment [32, 33].

For a long time, cell senescence has been considered as an anti-cancer phenomenon, while new findings reveal that senescent cells may have a dual activity by either inhibiting or promoting cancer growth or at least be ineffective in arresting tumor growth [34–36]. In general, the p53/p21^WAF1/CIP1^ pathway [37, 38] and/or the p16^INK4a^/pRb pathway are two main pathways for cellular senescence induction in cancerous tissues [39, 40]. However, it seems that in PCa additional pathways can be activated by therapy.

## Therapy-induced cellular senescence in PCa

Accumulating data indicate that the exposure of PCa to different anticancer compounds, ionizing radiation, and selected AR ligands induce a senescent phenotype, which is referred to as therapy-induced cell senescence (TIS). Importantly, recent studies suggest that the induction of cellular senescence as a cancer treatment may have beneficial effects for the patient [41–43]. Since TIS, in opposite to cell death, is mostly initiated at low dose of anticancer treatments, it might reduce the toxicity-related side effects and prolong patient survival [8, 44–46]. Further, it has been shown that the innate immune response is activated during senescence through upregulation of inflammatory cytokines which targets tumor cells and therefore have beneficial effects [47–49]. Thus the senescence program represents an alternative mechanism to prevent tumor growth of cancer cells that bypass many anti-proliferative pathways but are still sensitive to activation of a senescence program [41, 44].

However, on the other hand it has been reported that senescent cells may also promote tumor growth. Important to mention are the tumor-promoting properties of SASP including chronic inflammation, angiogenesis, stemness, migration and invasion [49, 50]. Therefore, TIS is an important determinant of therapy response for the clinical outcome for patients and rather under-investigated. Understanding the exact mechanisms and effects of TIS in PCa may help to prevent therapy resistances and prolong the survival of patients.

### Radiation-induced cellular senescence

One of the main used strategies for localized cancer treatment is radiotherapy or radiation therapy (RT). Nearly 50% of patients during their disease undergo radiation therapy. For many cancers, radiation is the primary treatment and can also be considered as neo-adjuvant or adjuvant with other treatments like chemotherapy [51]. RT exists in two forms based on the radiation source, internal treatment or radionuclide implants and external treatment or Linear Energy Transfer [52]. RT is based on high energy sources of radiation, like gamma and X-ray irradiations, electrons or protons. It is broadly considered as a treatment for PCa but around 30% of patients show disease recurrence [52]. Tumor RT changes sensitivity, viability and activity of cells and alters tumor microenvironment [53]. Another potential effect of RT in cancer cells is induction of cellular senescence [51, 53]. Gamma irradiation (2–75 Gy) on primary prostate epithelial cells from benign prostatic hyperplasia (BPH) or PCa showed diminution in colony-forming capability while, no more than 20% effect on cell viability. In line with this, gamma irradiation inhibits cell growth by inducing cellular senescence rather than apoptosis (Table 1) [52].

**Table 1:** Treatments that induce cellular senescence in PCa with indicated mechanisms

Therapy/treatment	Dose	Model system	Mechanism	References
Chemotherapy				
Docetaxel	5 nM	LNCaP	Anti-microtubule agent	[116]
Doxorubicin	25 nM	DU145/LNCaP	Anthracyclines, DNA damage	[116]
Mitoxantrone		BPH-1, RWPE-1, and PC3	Anthracyclines, DNA damage, p16^INK4a^, p21^WAF1/CIP1^	[32]
5-azacytidine	0.375 µM/75 µM	DU145/LNCaP	Antimetabolite, inhibition of DNA methyltransferase	[116]
Diaziquone	10 µM	DU145, LNCaP, PC3	DNA alkylation, p27^Kip1^	[115, 125]
Bithionol	10 µM	DU145, LNCaP	unknown	[115]
Dichlorophene	10 µM	DU145, LNCaP	unknown	[115]
Pyrithione	10 µM	DU145, LNCaP	Zn^2+^ ionophore, oxidative stress, ERK	[115]
Radiotherapy				
Gamma radiation	2–10 Gy	Primary prostate epithelial cells	DNA damage response	[52]
Gamma radiation	4 and 10 Gy	DU145, LNCaP, 22Rv1	DNA damage response	[56, 58]
X radiation	2, 4 and 8 Gy	PC3	P53, p21 ^WAF1/CIP1^, p16^INK4a^, p15^INK4b^	[55]
AR targeted therapy				
ADT		LNCaP, LAPC-4, LuCaP xenograft, PCa patient samples	p27^Kip1^, C/EBP β, oxidative stress, p16^INK4a^	[8, 67, 68]
AR agonist				
Dihydrotestosterone	10 nM	PC3, LNCaP, C4-2 PCa patient samples	p16^INK4a^, p21^WAF1/CIP1^, Cyclin D1, pRb,	[86, 184]
Methyltrienolone	1 nM	PC3, LNCaP, C4-2 PCa patient samples	p16^INK4a^, p21^WAF1/CIP1^, p27^Kip1^, Cyclin D1, E2F1, pRb, Src, AKT	[86, 90]
AR antagonists				
Bicalutamide	0.5–100 µM	LNCaP, CWR22PC	p16^INK4a^, p27^Kip1^	[68, 92]
Enzalutamide	10 µM	LNCaP, C4-2, LNCaP and C4-2 xenograft	p16^INK4a^	[88, 94, 185]
Darolutamide	10 µM	LNCaP, C4-2	p16^INK4a^	[94]
Atraric acid	1- 30 µM	LNCaP, C4-2 xenograft PCa patient samples	p16^INK4a^, pRb, Src, AKT	[91, 101]
20-aminosteroid (C18)	10 µM	LNCaP	Unknown	[95]
Anthranilic acid ester (C28)	30 µM	LNCaP	Unknown	[102]

**Fig. 1:**
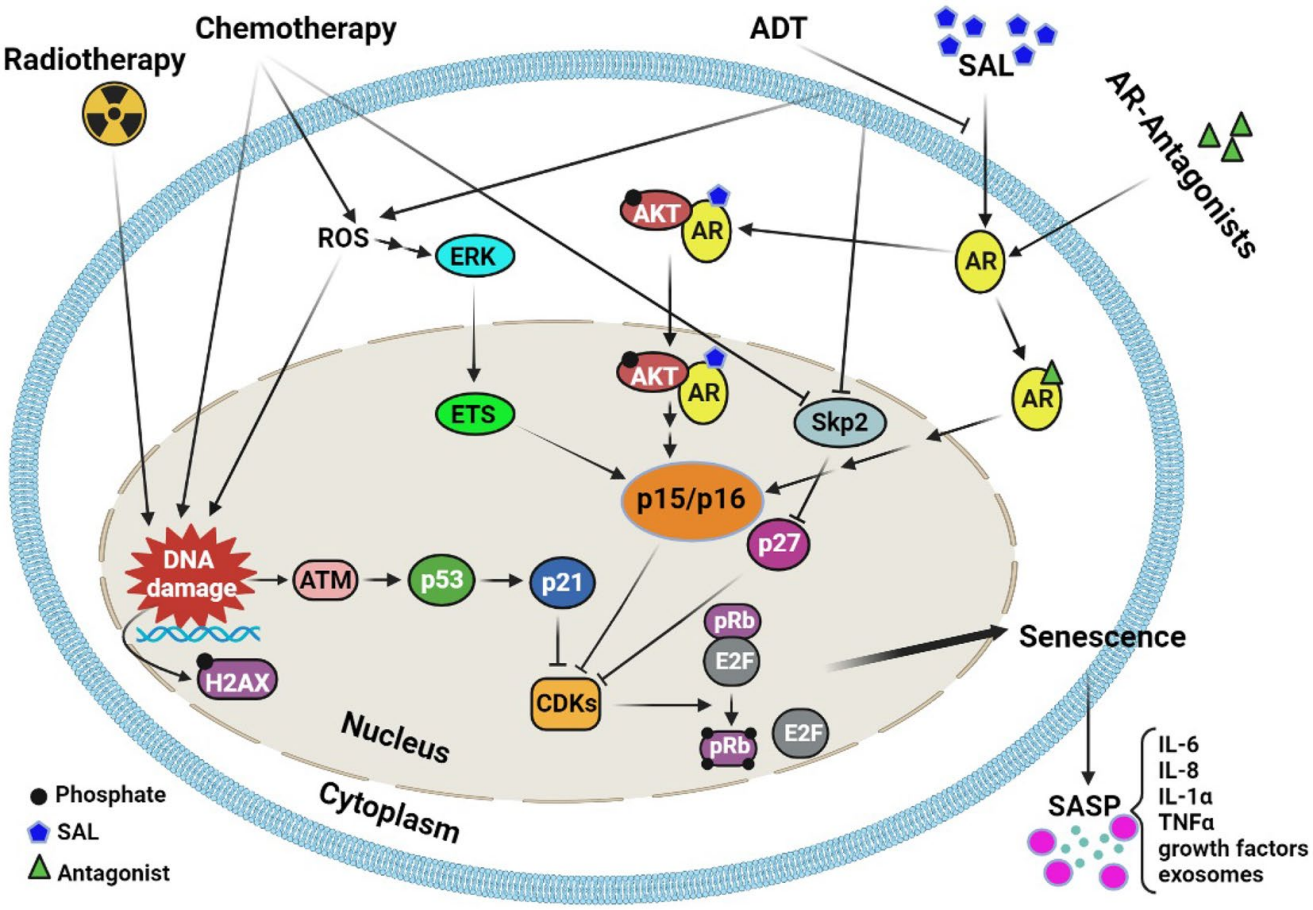
Molecular pathways of senescence-inducing therapies of PCa. Several applied therapies including radiotherapy, chemotherapy and androgen receptor (AR) targeted therapy (androgen deprivation therapy, ADT; supraphysiological androgen level, SAL; AR antagonists). Radiotherapy and chemotherapy lead to persistent DNA damage which triggers ATM or ATR signaling and finally p53 and p21^WAF1/CIP1^ activation. p21^WAF1/CIP1^ inhibits CDKs and mediates senescence through hypophosphorylation of pRb. In addition, chemotherapy induces senescence through ROS-ERK-ETS-p16^INK4a^ and the p27^Kip1^-pRb pathway. SAL mediates senescence by the p15^INK4b^-p16^INK4a^-pRb-E2F1 pathway. AR antagonists induce senescence through p15^INK4b^-p16^INK4a^. Senescent cells secrete many cytokines, growth factors and exosomes, known as senescence-associated secretory phenotype (SASP). These factors exert different autocrine/paracrine effects on the surrounding tumor microenvironment thereby promoting or inhibiting tumor growth (created in Biorender.com)

One of the predominantly activated factor after RT is the p53 tumor suppressor protein, which becomes activated in response to DNA double strand breaks (Fig. 1). p53 activation leads to cell cycle arrest, and can mediate either apoptosis or cellular senescence. The human PCa cell lines DU145 and PC3 are resistant to irradiation and have pairs of p53 inactivated alleles that cause p53 loss of function. On the other hand, the human PCa cell lines LNCaP and 22Rv1 with at least one wild type p53 allele are sensitive to RT with a similar sensitivity [54, 55] suggesting that TIS by radiation is mediated through p53.

In general, genetic alterations often are in line with increase RT resistance. Because DU145 cells are radio-resistant, few of them in response to irradiation go to either apoptosis or cellular senescence. By using 4 Gy of irradiation within 5 days only 5% of DU145 cells (p53mut/mut) show SA β-gal activity, while a similar dose of irradiation leads to induction of SA β-gal activity around 75% or 50% in LNCaP (p53wt/wt) and 22Rv1 (p53wt/mut), respectively. A high dose of irradiation, however, induces up to 30% cellular senescence in DU145 [56] indicating that the TIS pathway can also be activated through an p53-independent mechanism [56]. Interestingly, DU145 has a truncated, non-functional pocket protein pRb and lacks functional p53, the expression of a p53 transgene in this cell line is sufficient to express irradiation-induced senescence phenotype. Suggesting, some other factors like pocket protein family p107/p130 act downstream of p53-induced senescence [54, 57]. In addition, overexpression of dominant p53 mutant in LNCaP blocks and in 22Rv1 reduces the cellular senescence phenotype induced by irradiation [54].

In line with the RT-induced cell senescence, treatment with Nutlin-3, a small molecule compound that acts by disrupting the p53-MDM2 interaction and thus p53 stabilization to enhance tumor suppression, at concentrations of 5–10 µM efficiently sensitize PCa cells to clinically-relevant 2 Gy dose of irradiation by induction p53-dependent mediated cellular senescence [54, 58]. In 22Rv1 cell line 24 h after irradiation, p53 protein level still remained elevated. p21^WAF1/CIP1^ protein response to p53 was delayed but was elevated for at least 5 days. In addition, it has been suggested that the protein kinase ATM, as a DNA damage response, could phosphorylate p53 and leads to the stabilization of p53 expressed by 22Rv1 cells and subsequently transactivates its target gene p21^WAF1/CIP1^ [54]. Although the genomic integrity and clonogenic survival of 22Rv1 cells may be affected by the delayed transactivation of p21^WAF1/CIP1^, parental 22Rv1 cells did not significantly differ in radio sensitivity. Thus, taken together, it emerged that p53 activation mediates induction of cellular senescence in PCa in response to RT (Table 1) [54].

Interestingly, the compound resveratrol sensitizes p53-negative PCa cells to RT. Resveratrol (trans-3, 4, 5-trihydroxystilbene) is a polyphenolic compound that is naturally found in grapes. Multiple studies have shown different functions of resveratrol including neuroprotective, immunomodulatory, anti-inflammatory, antioxidant, and antitumor [59–61]. Resveratrol potentially synergizes with RT to inhibit PC3 cell proliferation and cell survival [55]. The percentage of PC3 colonies were decreased in a Gy-dependent manner. Treatment with resveratrol alone, decreases also percentage of PC3 colonies in a dose-dependent manner (2–50 µM) as well. Interestingly, after combined treatment with both RT and resveratrol (50 µM), cell proliferation and PC3 colonies were decreased synergistically. Thus, it suggests resveratrol strongly sensitizes PC3 cells to RT. In line with this, PC3 cells treated with 50 µM resveratrol and 8 Gy showed high mRNA level of p15^INK4b^, a senescence marker, and decreased Cyclin B and Cdk2. Although mRNA level encoding Cyclin D was increased in individually treated cells. The combined treatment resulted in reduced Cyclin D expression. Further, p-H2A.X as another marker of cellular senescence was significantly higher in the double treatment. These data suggest that cellular senescence may be the underlying mechanism for inhibition of both growth and clonogeneity resulting in synergic effect of resveratrol treatment with RT [21, 55].

Interestingly, synergistic induction of cellular senescence pathway have also been revealed for the combination treatment using radiation and Poly (ADP-ribose) polymerase (PARP) inhibitors [62]. PARP inhibitors have been approved for the treatment of PCa [63, 64]. Furthermore, it has been shown that the PARP inhibitor rucaparib increased radio-sensitization of PCa cell lines [62]. Following the combination treatment, PC3 and DU145 cell lines displayed abundant senescent cells [65, 66] indicating that RT sensitization and cellular senescence are enhanced by PARP inhibition in PC3 and DU145 cells.

### Androgen-deprivation induced cellular senescence

ADT can arrest cell proliferation and induce apoptosis in only a subpopulation of androgen-dependent PCa cells [67]. Tumor cells that fail to endure cell death may develop a senescence-like growth arrest both in vitro and in vivo [68, 69]. While apoptosis reaches a maximum within 72 h after androgen deprivation (AD), tumor cell senescence requires 3–6 days to acquire [41, 70]. In line with these findings, in vitro studies demonstrated that different PCa cell lines including LNCaP and LAPC-4 undergo senescence in response to culture in charcoal-stripped serum (CSS), which depletes for androgens and other steroids, thyroid and vitamin D3 hormones [67]. Under these conditions, more than 50% of the cell population showed senescent features after 7 days, while the percentage increased to more than 80% after 10 days culture in CSS. In contrast, the AR-negative and androgen-independent PCa cell line PC3 did not undergo senescence under AD. Consistently, PCa cells require AR signaling for the transition from G1 to S phase, based on this it is suggested that AD leads to proliferation arrest [71, 72].

The molecular markers of AD-induced senescence are a G1/S block, hypophosphorylated pRb, positive SA β-Gal activity staining, development of SAHF, nuclear expression of HP1γ, which alters the chromatin structure and therefore regulates gene expression in senescent cancer cells [67]. Further an increased expression of SASP that may contain insulin-like growth factor-binding protein 3 (IGFBP3) [73] and cathepsin B has been reported [67].

Mechanistically, ADT-induced senescence is partially mediated by the cyclin-dependent kinase inhibitor p27^Kip1^, which might depend on Skp2 (Table 1) [74]. Moreover, the transcription factor CCAAT/enhancer binding protein (C/EBP β) is upregulated in androgen-dependent PCa cells upon AD and is required for the development of a senescent phenotype [75]. C/EBP β stimulates the transcription of the senescence- associated factors IL-6/IL-8, decreases the expression of several E2F target genes and is a part of the pRb-E2F-dependent senescence [76, 77]. In addition, Burton et al. suggested the induction of senescence through AD by ROS-induced DNA damage and the p16^INK4a^ pathway in cell culture and human tumor-derived prostate tissue [68]. In this study the levels of p53 and p21^WAF1/CIP1^ were even decreased upon prolonged CSS culture suggesting that the AD-induced senescence is not mediated through the p53-p21^WAF1/CIP1^ pathway rather by ROS-induced DNA damage response and increase of p16^INK4a^ or the Skp2-p27^Kip1^-pRb pathway (Fig. 1).

Evidence suggest that AD treatment of cell lines selects for CRPC [78]. Importantly, cells cultured under AD for 3 days were able to resume proliferation in androgen-repleted media, while no proliferation was detected when cells were changed back to FBS media after 14 days in CSS media.

Moreover, a small population of cells (~ 0.1%) are capable to form colonies in CSS media after 3–4 weeks culture. Supporting these results, some weeks of cyclic/repeated exposure to androgen deprivation conditions facilitate the outgrowth of senescence-resistant androgen refractory LNCaP and LAPC-4 variants [68]. These subpopulations exhibit characteristics of clinical castration resistance including enhanced survivin and AR levels as well as TAp63, a prostatic basal stem cell marker [79, 80]. Hence, these data suggest that AD-induced senescence is associated with tumor progression and may promote CRPC development and chemoresistance through escape of cells from senescence. This occurs by cell autonomous-reprogramming and by the formation of a pro-tumorigenic SASP [68, 81].

Further evidence was provided by in vivo studies using LuCaP xenograft tumors in castrated nude mice revealing that AD increased SA β-Gal activity, increased expression of both p27^Kip1^ and HP1γ and decreased expression of the proliferation marker Ki-67 with minimal apoptosis. Similar results of increased senescence were observed in prostate tumors from patients that received AD therapy before radical prostatectomy [67].

In addition, senescent cells accumulate in PCa tissues over a prolonged time in patients following neoadjuvant ADT [8]. Notably lysosomal β-Galactosidase (GLB1) protein levels, the protein encoded by the *galactosidase beta 1* gene, increase in PCa patient samples during 6 months after ADT initiation [8]. Importantly, increased *GLB1* mRNA has been reported as a marker for improved outcomes in PCa [82], which suggests that PCa senescence is beneficial.

Thus, the overall consequence of senescent PCa cells remains inconclusive. PCa senescent cells through SASP may mediate within the tumor microenvironment resistance mechanisms that can lead to development of castration-resistant and drug-resistant tumors [26, 83] but AD-induced TIS seems to be beneficial for patients.

### AR signaling or AR ligand induced senescence

#### AR agonist induced cellular senescence

The bipolar androgen therapy (BAT) is a novel and at the first view a paradox approach used in clinical trials, treating patients with metastatic CRPC [84]. BAT consists of periodical oscillation between castration levels and supraphysiological levels of testosterone. High doses of androgens were observed in cell lines and in mouse xenograft model system to inhibit in an AR-dependent manner the growth of PCa [85]. This is in line with the biphasic androgen response of proliferation of PCa cells. While low androgen levels increase supraphysiological androgen level (SAL) decrease PCa cell proliferation [86]. BAT was also considered as an option to prevent the adaptation of PCa cells to a low-androgen level [87]. AR agonists, including dihydrotestosterone or the synthetic methyltrienolone at SAL, induce cellular senescence [86, 88, 89]. Interestingly, SAL treatment induces cellular senescence in various PCa model systems including CSPC, CRPC cell lines, 3D-PCa spheroids, as well as in native patient-derived PCa tissues (tumoroids) [86, 89].

SAL induced cellular senescence is AR-dependent and leads to upregulation of p16^INK4a^ and p15^INK4b^ and accordingly to hypophosphorylation of pRb. Reducing pRb phosphorylation targets E2F protein to downregulate E2F target genes such as *CCND1* encoding Cyclin D1 [86]. Hypophosphorylated pRb will inhibit E2F1-mediated transcriptional activity and thus progression to S-phase. siRNA -mediated knockdown of either p16^INK4a^ or p15^INK4b^ reduces the level of senescent cells, suggesting that, the p15^INK4b^-p16^INK4a^-pRb-E2F1 pathway (Table 1) regulates androgen-mediated cellular senescence in PCa cells [86, 90].

Interestingly, SAL enhances phospho-AKT (p-AKT) and phospho-S6 (p-S6) levels at a non-genomic level. Latter is a downstream target of AKT [88, 90]. Notably, the AKT inhibitor, AKTi, inhibits SAL-mediated cell senescence in PCa cell lines [91], suggesting that the non-genomic AR-AKT signaling mediates androgen-induced cellular senescence [90]. Since the knockdown of p15^INK4b^ reduces SAL-mediated cellular senescence but did not repress SAL-induced phosphorylation of AKT, revealing that the AR-AKT interaction is upstream of p15^INK4b^ in the SAL-induced senescence pathway (Fig. 1) [90]. RNA-seq data suggest a gene set that is associated and may control the SAL-mediated cellular senescence. Interestingly, one long non-coding RNA (LncRNA) was identified, the *lncRNASAT1* that is potently upregulated by SAL in both PCa cell lines and in SAL-treated native patient tumoroids [90]. Surprisingly, the knockdown of *lncRNASAT1* suppressed phosphorylation of AKT at S473 upon SAL treatment and reduced SAL-mediated cellular senescence, suggesting that the *lncRNASAT1* signaling is upstream of AKT [90]. Thus, AR-*lncRNASAT1*-AKT-p15^INK4b^ is a novel axis to mediate SAL-induced cellular senescence [90].

#### AR antagonists induced cellular senescence

It is noteworthy that cellular senescence in PCa cells can also be induced by treatment with the non-steroidal AR antagonists, Bicalutamide, atraric acid, enzalutamide, and darolutamide (Table 1) [92–94].

Also, amino-steroidal AR antagonists induce cellular senescence suggesting that in addition to SAL treatment inhibiting AR-mediated transactivation by AR antagonist induce cellular senescence in an AR-dependent manner. The data suggest that AR antagonist are not inactivating all AR signaling pathways rather antagonists induce the cellular senescence program [95].

Bicalutamide is one of the first-generation antagonists. This antagonist binds to the ligand binding domain of AR and ameliorates progression-free survival. Bicalutamide, by increasing the level of CDK inhibitors p16^INK4a^ and p27^Kip1^, induces cellular senescence [92, 93].

Enzalutamide, as a member of second-generation AR antagonist, blocks AR-androgen interaction and inhibits translocation of AR to the nucleus, thus reducing interaction between AR and DNA. Enzalutamide binds to AR with a 5–eightfold higher affinity compared to bicalutamide [93, 96, 97]. Enzalutamide arrests cell proliferation and induces cellular senescence in PCa [88]. Induction of cellular senescence by enzalutamide is accompanied by p16^INK4a^ induction. Enzalutamide treatment in combination with RT highly induces cellular senescence detected by SA β-gal staining in androgen dependent PCa cells (LNCaP and PC3-AR-T877A) compared to single treatments, while this induction is not significant in wild type AR-negative PCa cells (PC3 and PC3-AR-V7). Thus, enzalutamide radio-sensitizes PCa cells by induction of irradiation-dependent cellular senescence in AR expressing cells [98, 99].

Darolutamide is a second generation of AR antagonists. Similar to enzalutamide, darolutamide also upregulates p16^INK4a^ in both LNCaP and C4-2 cells, causing cellular senescence [15, 94, 100].

Atraric acid, a natural AR antagonist, inhibits proliferation and induces cellular senescence in cell culture, in both androgen dependent (LNCaP) and castration resistant (C4-2) PCa cells, as well as ex vivo in human PCa tumoroids derived from prostatectomies [91, 101]. Interestingly, atraric acid inhibits both wild-type and of those AR mutants that mediate resistance to AR antagonists such as bicalutamide and enzalutamide [101]. Atraric acid inhibits AR transactivation and increases cytosolic localized AR. Treatment of LNCaP cells with the combination of PP2, as a Src inhibitor, and atraric acid reduces cellular senescence in a dose-dependent manner. In addition, co-treatment of LNCaP cells with atraric acid and an AKT inhibitor reduces level of cellular senescence as well. Suggesting, that atraric acid induces cellular senescence by the AR-Src and AR-AKT interaction. The p53-p21^WAF1/CIP1^ signaling pathway after using atraric acid antagonist mostly remained without changes while, induction of p16^INK4a^ expression and down-regulation of pRb, E2F, Cyclin D1 were detected. In addition, after treatment of prostatectomy samples, in tumor samples SA β-gal activity and also induction of p16^INK4a^ but not p21^WAF1/CIP1^ mRNA level were detectable. Taken together, the data suggest that the p16^INK4a^ -pRb- E2F1 CyclinD1signaling pathway mediates atraric acid -induced cellular senescence [91].

Anthranilic acid esters are specific AR antagonists [102]. One anthranilic acid ester derivatives is C28 which mediates inhibition of AR transactivation, nuclear translocation and also induces cellular senescence [102]. Suggesting, cellular senescence is mediated by various AR antagonists and does not rely on specific antagonist structure.

### Chemotherapy-induced cellular senescence

Despite many progresses in treatment options, the main challenge of patients with metastatic, hormone-refractory disease is the limited survival of approximately 12 months and missing therapy options that prolong the survival of these patients [103, 104]. The combination of chemotherapy with hormone therapy or other therapies emerged as an effective strategy for men with symptomatic hormone-refractory PCa to reduce pain, increase quality of life and improve response rates [105, 106]. Recent studies investigated the combination of enzalutamide with the chemotherapy docetaxel in men with metastatic CRPC resulting in reduced PSA levels and improved patient outcomes [19, 20]. Moreover, docetaxel combined with ADT [107] shows extended overall survival compared to ADT alone [108, 109]. This indicates that combination treatments have the potential to be beneficial.

Most cancer cells undergo growth arrest or cell death in response to chemotherapy [110]. The induction of cellular damage by anticancer drugs causes initially a transient growth arrest [111, 112]. However, after drug removal some of the cancer cells, that survived the treatment, regain their proliferative capacity or die by mitotic catastrophe [113]. Notably, a small population of the cancer cells undergo prolonged growth arrest with features of senescence [114]. Numerous evidences suggest that high doses of chemotherapeutic drugs lead to cell death, whereas low doses of drugs show rather a more pronounced cytostatic effect, which presumably reflect the amount of DNA damage [114]. In contrast to cell death, the drug-induced senescence requires several days to establish [41].

Accumulating data demonstrate that selected chemotherapeutic drugs induce permanent growth arrest with phenotypic characteristics of cell senescence in PCa [114–116]. These drugs use different mechanisms to initiate senescence involving DNA damage, oxidative stress, and DNA methylation alterations [117]. It has been shown that DNA damaging agents such as doxorubicin are more effective in inducing senescence in vitro than microtubule targeting agents like docetaxel [118].

Docetaxel at a dose of 5 nM initiates senescence features in the androgen-dependent LNCaP cell line (AR positive and wild-type p53) evident by morphological changes, proliferative failure and multinucleation [116]. However, docetaxel did not induce a senescent phenotype in hormone-refractory DU145 cells (AR negative and mutant p53) at any dose. However, both DU145 and LNCaP cells develop a senescence-like phenotype when treated with low doses of doxorubicin (25 nM) for 3 days [116]. On the other hand, increased concentrations of doxorubicin (100 nM and 250 nM) induced apoptosis [115]. Doxorubicin belongs to the family of anthracyclines, which intercalate into DNA and inhibit topoisomerase II leading to inhibition of DNA and RNA synthesis [119, 120]. The detailed underlying mechanism by which doxorubicin induces cellular senescence has not yet been investigated.

However, the effect of doxorubicin-induced senescent cells in the microenvironment has been analyzed. Interestingly, doxorubicin-induced senescent cancer cells increase the proliferation of co-cultured cells in vitro by paracrine signaling [121]*.* However, this proliferative bystander effect was significant less compared to coculture with senescent fibroblast indicating different effects in the environment. In contrast, senescent cancer cells did not increase proliferation and growth of LNCaP and DU145 xenografts in nude mice [121]. It has been shown that tumors were even smaller in the presence of senescent cells, which persisted 5 weeks in the tumor suggesting that the induction of senescence in PCa would not promote tumor growth [121].

Different markers that are upregulated during drug-induced senescence (docetaxel, doxorubicin and 5-azacytidine) in LNCaP and DU145 were identified including *CSPG2*, *CXCL14, Adlican* and *COL1A1* [116]. Importantly, some of the upregulated genes have pro-tumorigenic effects, while others may inhibit tumor growth. Studies found increased levels of the protease CSPG2 in the peritumor extracellular matrix of breast cancers, which suggest that CSPG2 promotes tumor invasion [122].

However, overexpression of the chemokine CXCL14 inhibits the growth of LAPC4 xenografts suggesting a tumor-suppressive role in PCa [123]. It still needs to be elucidated which effects chemotherapy-induced genes have for the progression of PCa. Since these senescence-induced genes are also upregulated in p53 deficient cells, it seems that the induction of senescence by chemotherapeutics is effective independent of the p53 status [41, 114].

Mitoxantrone is another member of the anthracyclines that is used to treat PCa and has been combined with prednisone to reduce pain and improve the quality of life for patients with advanced, hormone-refractory PCa [105]. This drug induces SASP factors and increases the mRNA expression of the senescence markers p16^INK4a^ and p21^WAF1/CIP1^ in three different prostate epithelial cancer cell lines BPH-1, RWPE-1, and PC3 and prostatectomy samples from PCa patients [32].

Another class of anticancer drugs that induce senescence through alterations in the DNA structure and function are antimetabolites. 5-azacytidine belongs to this class and functions at low concentrations as DNA methyltransferase inhibitor. Continuously treated DU145 and LNCaP cells become senescent within 7 days indicating that epigenetic changes can lead to cellular senescence [116].

Ewald et al. developed a high-throughput method to identify new compounds that induce senescence in PCa cell lines. The four lead compounds diaziquone, bithionol, dichlorophene, and pyrithione induce a persistent growth arrest associated with increased SA β-Gal staining and elevated expression of the senescence marker genes *Glb1* (encodes beta-galactosidase-1), *BRAK* and *CSPG2*. Diaziquone is an DNA alkylating agent with a broad antitumor activity including transplantable murine tumors [124]. Interestingly, the cyclin dependent kinase inhibitor p27^Kip1^ is induced by diaziquone in LNCaP, PC3 and DU145 cells (Table 1). The negative regulator of p27^Kip1^, the ubiquitin-ligase Skp2, is an important regulator of the TIS, since overexpression of the ligase inhibits the effects of diaziquone on senescence and the induction of p27^Kip1^ [125]. In line with these findings, an elevated Skp2 expression is associated with a poor prognosis in PCa [126].

Pyrithione is a Zn^2+^ ionophore that generates oxidative stress through ERK activation (Fig. 1) leading to growth arrest [127, 128]. Yet, it remains unknown how bithionol and dichlorophene induce senescence [115].

In conclusion, TIS should be considered exhibiting both beneficial and also adverse effects for the patient. A major positive effect of senescence relies on the growth inhibition of target cells, which restrict the disease progression [42]. Moreover, studies reported the spreading of senescence towards neighboring cancer cells suggesting tumor-suppressive effects [45, 129]. However, TIS might also promote tumor progression with SASP being presumably an important factor.

## Effects of senescent cells on the tumor microenvironment

### Therapy-induced SASP

In general, senescent cells secrete several inflammatory cytokines, such as IL6 and IL8 also termed CXCL8, [26, 130], chemokines, growth factors and proteases (Fig. 1). Senescent cells become apoptosis-resistant through upregulation of the Bcl-2 antiapoptotic protein family and other factors [25, 26]. SASP can alter the tissue microenvironment [130], contributing to age-related pathologies [131, 132]. It can agitate the structure and function of normal tissues and promote malignant phenotypes in nearby cells [133, 134]. Although cellular senescence is generally considered as a natural tumor suppressor mechanism, senescent prostate fibroblasts populating aged tissue, may secrete soluble growth factors, which are able to change the microenvironment and affect also non-senescent fibroblasts [133]. In addition, senescence of aged normal prostate contributes to prostate tissue growth leading to BPH [135]. Senescent fibroblasts are metabolically active and release high levels of epithelial growth factors and matrix metalloproteinases [136, 137]. Thus, an accumulation of senescent fibroblasts can alter the surrounding microenvironment; promote growth and tumor progression of initiated human prostate epithelial cells [138, 139].

Therapy-induced SASP, such as by chemotherapy and ionizing radiation, develops from an acute stress-associated phenotype and usually arises 5 to 8 days after the onset of treatment in PCa [140, 141]. Acute stress-associated phenotype in senescent cells is a relatively rapid cellular response to cytotoxic agents. It is secreted 24 h after treatment and before the appearance of the senescence markers SASP [141]. Similarly, induction of cellular senescence by DNA damage treatment like bleomycin, mitoxantrone, radiation and non-DNA damage treatment including docetaxel, paclitaxel, vincristine of the primary prostate fibroblast cell line PSC27 showed that p38, a stress-inducible mitogen-activated protein kinase (MAPK), is mostly activated by DNA damage treatment rather than by non-DNA damage treatment [141]. Notably, p38 regulates the SASP in normal human fibroblasts via the PI3K/AKT/mTOR pathway [132]. In addition, it was also demonstrated that the ATM-TRAF6-TAK1 axis is formed rapidly after genotoxic treatment in PSC27 cells. TAK1 phosphorylates p38, occurring at a time between the acute stress-associated phenotype and the expression of SASP [141].

Another important regulator of SASP is epigenetic regulator lysine demethylase KDM4. KDM4 belongs to the demethylase subfamily, that target histone H3 on lysine 9 and 36 positions and thereby change chromatin remodeling [142]. Decreased methylation of H3K9/H3K36 and upregulation of KDM4 are correlated with poor survival of PCa patients after chemotherapy [143]. It has been shown that treatment of PSC27 cells with different chemotherapeutic agents such as cisplatin, carboplatin, satraplatin, mitoxantrone and doxorubicin induced the KDM4 family members and increased senescence markers including p16^INK4a^, p21^WAF1/CIP1^and the particular SASP factor CXCL8 [143]. Consistently, similar results were observed in biospecimens of PCa patients after chemotherapy. In line with this, by using the KDM4 inhibitor ML324 the expression of CXCL8, CSF2, CCL20, IL-1α, CXCl1 and IL-6, as SASP factors, reduced at mRNA and protein levels with unchanged senescence markers [143]. It suggests that KDM4 inhibition affects the SASP by epigenomic remodeling while retaining cellular senescence.

IL-1α is considered as a SASP factor. Interestingly, IL-1α binds to its cell surface receptor (IL1R1) on senescent cells but is not secreted by senescent cells and thus may not impose effects on neighbor cells in the microenvironment. However, its abundance on senescent cells surface increases significantly in the presence of senescence stimuli, where it plays a key role in establishing and maintaining the SASP [76].

### Exosomes

In addition, senescent cells secrete small extracellular vesicles (sEVs) [144]. sEVs are heterogeneous populations of membrane vesicles [145], including exosomes and exomeres [146]. Exosomes are surrounded by a phospholipid bilayer. Their size range is approximately 30–100 nm [147]. Exosomes derive from the intraluminal vesicles in late endosomal compartments by the inward budding of the endosomal membranes and are released from cells upon fusion of the outer membrane of late endosomal membrane with the plasma membrane [148]. Most cells are able to secrete exosomes (Fig. 1). They carry components, such as proteins, mRNA, microRNA, lncRNA, circRNA, and DNA [149]. Proteins that are found in exosomes include membrane transporters such as annexins, flotillins, GTPases, heat shock proteins, tetraspanins, lactadherin, platelet-derived growth factor receptor, transmembrane proteins, lipid-related proteins and phospholipases [150]. It is important to emphasize that the exosomes content varies between cells from which they originate. Thus, their content might be used as specific biomarkers of various diseases. Besides these specific markers, exosomes have common markers. Because of their endosomal origin, exosomes contain protein markers such as flotillin, CD9, CD63, etc., which are found in exosomes from different cells [150].

Exosomes play an important role in intercellular communication and signaling [151]. It is noteworthy that they are involved in regulating the tumor-normal cell communication in the tumor microenvironment [152]. They can impose different effects on recipient cells based on their content [153].

Cancer cells secrete exosomes, which may participate in modulating their signaling pathways associated with tumor promotion, immune escape, drug resistance, and anti-apoptotic features [154, 155]. Additionally, cancer cell-derived exosomes can stimulate apoptosis and consequently inhibit tumor growth. For example, exosomes secreted by HEK293, a human embryonic kidney cell line, and HT-1080, a fibrosarcoma cell line, can suppress growth and proliferation of p53-deficient cells [156]. Also, isolated exosomes from A549 senescent cells, lung carcinoma epithelial cells, with normal PTEN, delivered this factor to PTEN-deficient PC3 cells causes growth arrest [150].

Besides growth arrest, exosomes play an active role in PCa development and also therapy resistance such as docetaxel resistance [147]. Indeed, exosomes derived from PCa cells influence PCa progression, mesenchymal stem cell differentiation into pro-angiogenic and pro-invasive myofibroblasts [157], drug resistance [158], and bone metastasis. The functional properties of the exosome-generated myofibroblasts reinforce the hypothesis that cancer exosomes have a cancer-promoting influence [157]. Furthermore, PCa-derived exosomes can modulate osteoblast function in the bone metastatic niche, specifically due to their miRNAs [159, 160]. In more detail, exosomal miR-141-3p derived from MDA-PCa-2b cells regulates osteoblast activity and increases osteoprotegerin expression [159]. Also, exosomal hsa-miR-940 derived from C4-2 cells stimulates the osteogenic differentiation of human mesenchymal stem cells [161]. In addition, exosomes containing circRNAs not only regulate cancer progression but also affect chemosensitivity in human cancer [162]. For example, exosome-mediated circ-XIAP, circRNA X-linked inhibitor of apoptosis, enhances docetaxel resistance of PCa cell lines by regulating miR-1182/TPD52 axis because circ-XIAP directly targets miR-1182. Thus, circ-XIAP can be transported via exosomes into the microenvironment [147].

For diagnosis of AR-antagonists resistance, the exosomal AR splicing variant 7 (ARv7), and P-glycoprotein (P-gp) have been reported. ARv7 splice variant lacks the androgen binding domain and mediates resistance to androgen-targeted therapy. It is suggested that exosomal *ARv7* mRNA could be a marker to diagnose enzalutamide or abiraterone resistance [163]. P-gp encoded by the multidrug resistance protein 1 (*MDR1*) gene, acts as a drug efflux pump and contributes to the development of resistance against chemotherapy [164]. P-gp protein levels were not only present and detectable in exosomes secreted from docetaxel-resistant PCa cells (PC3-R) but higher levels were detected in exosomes isolated from the serum of patients with docetaxel-resistant cancers [165]. In addition, the *CD44v8-10* mRNA may be involved in docetaxel-resistance in PCa. Therefore, serum exosomal *CD44v8-10* mRNA could be considered as a diagnostic marker for docetaxel-resistant CRPC [166]. Beside *CD44v8-10* mRNA, prostate specific membrane antigen (PSMA) may serve as a marker. PSMA is a cell surface antigen highly expressed in prostate especially in advanced PCa compared to other organs [167]. PSMA could be an excellent target for the isolation of PCa-specific exosomes. Because PSMA is highly expressed in plasma of patients with advanced PCa and castration- and chemotherapy resistant PCa compared to plasma of healthy volunteers or PCa patients without metastasis. This suggests higher levels in exosomes [168]. Thus, PSMA is a good candidate to be a diagnostic marker and also help to isolate PCa-related exosomes more accurately. So far, it is unclear that exosomes in the tumor microenvironment can lead to cellular senescence in PCa.

## Senotherapeutics

Senescence induction benefits are debatable because there are increasing reports that specific SASP may cause tumors to grow [93]. Consequently, targeting specifically senescent tumor cells with senotherapeutics may be a useful strategy for PCa treatment [93]. Senotherapeutics reduce the proportion of senescent to non-senescent cells by either killing senescent cells specifically (senolytic) or by inhibiting part or all of their characteristics (senomorphics) [169, 170]. Senolytic compounds enhance the death of senescent cells by temporarily blocking the pro-survival/anti-apoptotic pathway activated in senescence cells. This includes the activation of PI3K/AKT and/or Bcl-2/Bcl-xl pathways, which are activated during the senescence process [28, 171].

Notably, senescent cells and cancer cells have the same trait of resistance to apoptosis. Thus, medications that target anti-apoptotic proteins, such as Navitoclax (ABT-263), ABT-737, and A1155463 (A-115), which inhibit Bcl-2 family members, exhibit senolytic characteristics [28, 31, 172, 173]. It was reported that ABT-263 and A-115 induce cell death in XRA-TIS PCa cells. In fact, treating LNCaP and PC3 cell lines with 0.625 µM ABT-263 or 0.3125 µM A-115 for 6 days after cellular senescence induction by a single dose of XRA (8 Gy) led to enhancement of cell death by up to 80%. It suggests that ABT-263 and A-115 can be used as senolytics to induce apoptosis in XRA-TIS PCa cell lines [21]. However, treatment with ABT-263 after inducing cellular senescence in LNCaP cells by either SAL [88] or enzalutamide [21, 88] does not increase the death rate of senescent cells. In addition, less cleaved PARP (c-PARP) was detected in the AR ligand-induced senescent LNCaP cells than the control-treated cells, indicating resistance to induction of apoptosis in the former one [88]. Thus, it can be implied that Bcl-2 family inhibitors are efficient to kill XRA-TIS cells but may not have any senolytic effect in AR ligand-induced senescent PCa cells [21, 88]. Interestingly, various senescence inducers may lead to senescent cells expressing a different elevated pro-survival/anti-apoptotic pathway. This might restrict the ability of a single senolytic drug to target only one of these routes selectively [88]. Therefore, it can be concluded that DNA damage, by XRA-TIS, is the key factor to sensitizing the Bcl-2 family senolytics [21].

The anti-apoptotic protein Myeloid cell leukemia-1 (Mcl-1) belongs to the Bcl-2 family which suppresses apoptosis [174]. Numerous tumor types typically exhibit overexpression of Mcl-1, which is strongly correlated with carcinogenesis, a poor prognosis, and medication resistance [175, 176]. When prostate cancer cells (PC3 and LNCaP) were treated with docetaxel and palbociclib, they became senescent, and using of S63845, a Mcl-1 inhibitor, reduced the percentage of SA β-gal positive cells. It leads to apoptosis of senescent cells and enhancement of cleaved caspase 3 [177]. As a result, S6345 can be suggested as another senolytic drug, which targets and inhibits Mcl-1 in PCa cell lines [176, 177].

In addition to the aforementioned compounds, HSP90 inhibitors like geldanamycin can also be considered as senolytic drugs. These kinds of inhibitors suppress the pro-survival AKT-pathway because HSP90 protects AKT from proteasomal degradation [178]. Pungsrinont et al. introduced Ganetespib, a HSP90 inhibitor, as a senolytic compound in pretreated PCa cells. It significantly enhanced apoptosis and c-PARP protein levels after SAL-induced cellular senescence but not after enzalutamide-induced cellular senescence in the LNCaP cell line [88]. Additionally, this inhibitor increased cell detachment and decreased the percentage of SA β-Gal positive cells following SAL-induced cellular senescence. It is proposed that AR antagonist-treated LNCaP cells are resistant to ganetespib-induced apoptosis and ganetespib has a high senolytic activity after SAL-treated cells [88]. Using MK2206, an AKT inhibitor, as a senolytic drug with AR antagonist-specificity leads to increased cell detachment and also a high level of c-PARP in LNCaP cell line indicating MK2206 as a senolytic for enzalutamide-induced cellular senescence. While MK2206 had the opposite effect on the AR agonist-induced senescent cells, it suggests that SAL can develop a defense mechanism against this inhibitor [88]. As a result, dependent on the kind of AR ligand, MK2206 or ganetespib revealed specific senolytic activity in PCa cell lines.

In addition to senolytic compounds, some SASP inhibitors are termed senomorphics. They can directly or indirectly attenuate the SASP of senescent cells by suppressing the transcription factor NF-κB, the JAK-STAT signal transduction pathway, the serine/threonine protein kinase mTOR, or other pathways that contribute to the initiation and maintenance of the SASP without cytotoxicity [178]. For instance, rapamycin and metformin as senomorphics lead to the reduction of SASP expression. Metformin weakens SASP effects by reducing the expression levels of pro-inflammatory cytokines mostly regulated by NF-κB, chemokines and serpin genes in the context of RAS-induced senescence in metformin-treated senescent cells [179]. In the PC3 cell line treated with conditioned medium derived from senescent fibroblast cells followed by metformin treatment, NF-κB function was suppressed by inhibiting its translocation into the nucleus, and IκB and IKKα/β phosphorylation were reduced [179]. Rapamycin is an mTORC1 complex inhibitor. IL-6 and IL-8 secretion was reduced under rapamycin treatment from senescent PSC27 adult prostate [180]. Furthermore, IL-1α protein levels but not its transcript were reduced in senescent cells [180], indicating a post-translational control in the presence of rapamycin. Notably, IL-1α reduction decreased transcriptional activity of NF-κB, which regulates a large portion of the SASP [132, 180]. As a consequence, it suggests that rapamycin and metformin reduce the stimulation of proliferation, migration, and invasion effects of SASP from senescent cells on nearby cells [180, 181].

Generally, senotherapeutics, among other emerging therapeutic options, may be able to slow the growth of PCa by eliminating senescent cells.

## Conclusion and further remarks

The major challenge in the treatment of metastatic PCa is the development of resistances to the existing therapies and progression to a disease state, which is still uncurable. Understanding the molecular mechanisms leading to disease progression are important to develop new treatment strategies for PCa. It is noteworthy that a number of treatments such as radiotherapy, AR targeted therapies (ADT, AR agonists, AR antagonists) and chemotherapy have been demonstrated to induce senescence in PCa. Hence, these standard treatments exhibit their anticancer effects not only through induction of apoptosis but by senescence as well. Important to note is that tumor cells that developed resistance mechanisms against apoptosis can be still targeted through senescence. Interestingly, the molecular pathways inducing the cell cycle arrest differ between the therapies. In PCa, TIS by AR ligands seems to activate the p15^INK4b^-p16^INK4a^-pRb signaling as a key pathway to induce cellular senescence [90, 91]. In contrast, the p53-p21^WAF1/CIP1^ pathway is the most detected during radiotherapy-induced senescence suggesting that the type of therapy induces a preferred TIS pathway. Moreover, in chemotherapy and ADT the ROS-ERK-ETS-p16^INK4a^ and the p27^Kip1^-pRb pathways are activated to induce TIS. In line with that PCa cells have different intracellular signaling to induce TIS.

One of the major challenges in this field is the identification of selective and pathway-specific senescence-inducing compounds and more reliable senescence markers.

It should be considered that TIS might have both, beneficial and adverse effects. Senescent cells activate the innate immune response, which target tumor cells and kill them. However senescent cells secrete also soluble inflammatory growth factors (SASP) and extracellular vesicles like exosomes, which change tumor microenvironment and might promote tumor growth. Studies demonstrated that the molecules of exosomes secreted by senescent cells are potential markers to diagnose drug resistance in PCa.

Still, the immediate and long-term effects of the treatment-induced senescent cells on the tumor microenvironment are poorly understood. Thus, there is a need to study the effects of these senescent cells on the disease progression of PCa in more detail. Recently senotherapeutics were introduced as new approach to target therapy-induced senescent cells. Further analysis of the clinical practice of senotherapeutics for PCa are necessary to validate the potential adverse effects for patients. The understanding of cellular senescence in cancer therapy remains an extensive field of research that contains many unexploited possibilities for the future.

Different treatments leading to TIS may activate differences in the composition of SASP. Thus, it is possible that some treatments may induce a stronger inflammatory signaling in the tumor microenvironment compared to others and will lead to different prognostics. Also, since various of types of senescence inducers are known, which may overlay the TIS such as dysfunctional mitochondria, epigenetic changes, genomic instability, reactive metabolites and oxidative stress and viral infection, the genetic program of senescence induction and SASP composition may differ. Artificial intelligence may be used to define the heterogeneity of cellular senescence levels in PCa tumors. A recent study analyzing cellular senescence of single cell profiles in the tumor microenvironment indicates associations with genomic and immune pathways that may enable predicted immunotherapy responses and patient prognosis including PCa [182]. Similarly, bioinformatic tools can be used to establish a cellular senescence-related gene prognostic index in order to predict metastasis and radio-resistance in PCa [183]. These predictions, however, must be verified to validate the link between senescence levels as biomarkers of PCa and patient prognosis that might be used in future as a basis for therapeutic interventions.

## Data Availability

Not applicable.

## Abbreviations

AD: Androgen deprivation
ADT: Androgen deprivation therapy
AR: Androgen receptor
BAT: Bipolar androgen therapy
BPH: Benign prostatic hyperplasia
C/EBP β: CCAAT/enhancer binding protein
CRPC: Castration resistant PCa
CSS: Charcoal-stripped serum
lncRNA: Long non-coding RNA
MAPK: Mitogen-activated protein kinase
Mcl1: Myeloid cell leukemia-1
MDR1: Multidrug resistance protein 1
PARP: Poly ADP-ribose polymerase
PCa: Prostate cancer
PSA: Prostate specific antigen
pRb: Phospho retinoblastoma protein
RT: Radiation therapy
SA β-gal: Senescence-associated beta galactosidase
sEVs: Small extracellular vesicles
SAHF: Senescence-associated heterochromatin foci
SAL: Supraphysiological androgen level
SASP: Senescence-associated secretory phenotype
TIS: Therapy-induced cellular senescence

## References

[1] Shen T, Wang W, Zhou W, Coleman I, Cai Q, Dong B (2021). MAPK4 promotes prostate cancer by concerted activation of androgen receptor and AKT. J Clin Investig.

[2] Mottet N, Bellmunt J, Bolla M, Briers E, Cumberbatch MG, De Santis M (2017). EAU-ESTRO-SIOG guidelines on prostate cancer. Part 1: screening, diagnosis, and local treatment with curative intent. Eur Urol.

[3] Park JW, Jang WS, Koh DH, Ham WS, Rha KH, Hong SJ (2018). Impact of early salvage androgen deprivation therapy in localized prostate cancer after radical prostatectomy: a propensity score matched analysis. Yonsei Med J.

[4] Sharifi N, Gulley JL, Dahut WL (2005). Androgen deprivation therapy for prostate cancer. JAMA.

[5] Huggins C, Hodges CV (1972). Studies on prostatic cancer. I. The effect of castration, of estrogen and androgen injection on serum phosphatases in metastatic carcinoma of the prostate. CA Cancer J Clin..

[6] Wong YNS, Ferraldeschi R, Attard G, de Bono J (2014). Evolution of androgen receptor targeted therapy for advanced prostate cancer. Nat Rev Clin Oncol.

[7] Liu H-H, Tsai Y-S, Lai C-L, Tang C-H, Lai C-H, Wu H-C (2014). Evolving personalized therapy for castration-resistant prostate cancer. Biomedicine.

[8] Blute ML, Damaschke N, Wagner J, Yang B, Gleave M, Fazli L (2017). Persistence of senescent prostate cancer cells following prolonged neoadjuvant androgen deprivation therapy. PLoS ONE.

[9] Perner S, Cronauer MV, Schrader AJ, Klocker H, Culig Z, Baniahmad A (2015). Adaptive responses of androgen receptor signaling in castration-resistant prostate cancer. Oncotarget.

[10] Dehm SM, Schmidt LJ, Heemers HV, Vessella RL, Tindall DJ (2008). Splicing of a novel androgen receptor exon generates a constitutively active androgen receptor that mediates prostate cancer therapy resistance. Can Res.

[11] Ehsani M, David FO, Baniahmad A (2021). Androgen receptor-dependent mechanisms mediating drug resistance in prostate cancer. Cancers.

[12] Nakazawa M, Paller C, Kyprianou N (2017). Mechanisms of therapeutic resistance in prostate cancer. Curr Oncol Rep.

[13] Pungsrinont T, Kallenbach J, Baniahmad A (2021). Role of PI3K-AKT-mTOR pathway as a pro-survival signaling and resistance-mediating mechanism to therapy of prostate cancer. Int J Mol Sci..

[14] Mottet N, De Santis M, Briers E, Bourke L, Gillessen S, Grummet JP (2018). Updated guidelines for metastatic hormone-sensitive prostate cancer: abiraterone acetate combined with castration is another standard. Eur Urol.

[15] Bastos DA, Antonarakis ES (2019). Darolutamide for castration-resistant prostate cancer. Onco Targets Ther.

[16] Nevedomskaya E, Baumgart SJ, Haendler B (2018). Recent advances in prostate cancer treatment and drug discovery. Int J Mol Sci.

[17] Rathkopf DE, Scher HI (2018). Apalutamide for the treatment of prostate cancer. Expert Rev Anticancer Ther.

[18] Rajaram P, Rivera A, Muthima K, Olveda N, Muchalski H, Chen QH (2020). Second-generation androgen receptor antagonists as hormonal therapeutics for three forms of prostate cancer. Molecules..

[19] Caffo O, Ortega C, Nolè F, Gasparro D, Mucciarini C, Aieta M (2021). Docetaxel and prednisone with or without enzalutamide as first-line treatment in patients with metastatic castration-resistant prostate cancer: CHEIRON, a randomised phase II trial. Eur J Cancer.

[20] Morris MJ, Rathkopf DE, Novotny W, Gibbons JA, Peterson AC, Khondker Z (2016). Phase Ib study of enzalutamide in combination with docetaxel in men with metastatic castration-resistant prostate cancer. Clin Cancer Res.

[21] Malaquin N, Vancayseele A, Gilbert S, Antenor-Habazac L, Olivier MA, Ait Ali Brahem Z (2020). DNA damage-but not enzalutamide-induced senescence in prostate cancer promotes senolytic Bcl-xL inhibitor sensitivity. Cells..

[22] Collado M, Gil J, Efeyan A, Guerra C, Schuhmacher AJ, Barradas M (2005). Senescence in premalignant tumours. Nature..

[23] Muñoz-Espín D, Cañamero M, Maraver A, Gómez-López G, Contreras J, Murillo-Cuesta S (2013). Programmed cell senescence during mammalian embryonic development. Cell.

[24] Storer M, Mas A, Robert-Moreno A, Pecoraro M, Ortells MC, Di Giacomo V (2013). Senescence is a developmental mechanism that contributes to embryonic growth and patterning. Cell.

[25] Alessio N, Aprile D, Squillaro T, Di Bernardo G, Finicelli M, Melone MA (2019). The senescence-associated secretory phenotype (SASP) from mesenchymal stromal cells impairs growth of immortalized prostate cells but has no effect on metastatic prostatic cancer cells. Aging.

[26] Carpenter V, Saleh T, Lee SM, Murray G, Reed J, Souers A (2021). Androgen-deprivation induced senescence in prostate cancer cells is permissive for the development of castration-resistance but susceptible to senolytic therapy. Biochem Pharmacol.

[27] Cuollo L, Antonangeli F, Santoni A, Soriani A (2020). The senescence-associated secretory phenotype (SASP) in the challenging future of cancer therapy and age-related diseases. Biology.

[28] Fiard G, Stavrinides V, Chambers ES, Heavey S, Freeman A, Ball R (2021). Cellular senescence as a possible link between prostate diseases of the ageing male. Nat Rev Urol.

[29] Özcan S, Alessio N, Acar MB, Mert E, Omerli F, Peluso G (2016). Unbiased analysis of senescence associated secretory phenotype (SASP) to identify common components following different genotoxic stresses. Aging.

[30] Saleh T, Tyutyunyk-Massey L, Murray GF, Alotaibi MR, Kawale AS, Elsayed Z (2019). Tumor cell escape from therapy-induced senescence. Biochem Pharmacol.

[31] Zhu Y, Tchkonia T, Fuhrmann-Stroissnigg H, Dai HM, Ling YY, Stout MB (2016). Identification of a novel senolytic agent, navitoclax, targeting the Bcl-2 family of anti-apoptotic factors. Aging Cell.

[32] Coppé JP, Patil CK, Rodier F, Sun Y, Muñoz DP, Goldstein J (2008). Senescence-associated secretory phenotypes reveal cell-nonautonomous functions of oncogenic RAS and the p53 tumor suppressor. PLoS Biol.

[33] Huda N, Liu G, Hong H, Yan S, Khambu B, Yin X-M (2019). Hepatic senescence, the good and the bad. World J Gastroenterol.

[34] Özcan S, Alessio N, Acar MB, Toprak G, Gönen ZB, Peluso G (2015). Myeloma cells can corrupt senescent mesenchymal stromal cells and impair their anti-tumor activity. Oncotarget.

[35] Schosserer M, Grillari J, Breitenbach M (2017). The dual role of cellular senescence in developing tumors and their response to cancer therapy. Front Oncol.

[36] Yang J, Liu M, Hong D, Zeng M, Zhang X (2021). The paradoxical role of cellular senescence in cancer. Front Cell Dev Biol..

[37] Zhang Y, Dong Y, Melkus MW, Yin S, Tang S-N, Jiang P (2018). Role of P53-senescence induction in suppression of LNCaP prostate cancer growth by cardiotonic compound bufalin. Mol Cancer Ther.

[38] Kang J-Y, Kim JJ, Jang SY, Bae Y-S (2009). The p53–p21Cip1/WAF1 pathway is necessary for cellular senescence induced by the inhibition of protein kinase CKII in human colon cancer cells. Mol Cells.

[39] Campisi J (2005). Senescent cells, tumor suppression, and organismal aging: good citizens, bad neighbors. Cell.

[40] Campisi J, d’Adda di Fagagna F (2007). Cellular senescence: when bad things happen to good cells. Nat Rev Mol Cell Biol..

[41] Ewald JA, Desotelle JA, Wilding G, Jarrard DF (2010). Therapy-induced senescence in cancer. J Natl Cancer Inst.

[42] Mikuła-Pietrasik J, Niklas A, Uruski P, Tykarski A, Książek K (2020). Mechanisms and significance of therapy-induced and spontaneous senescence of cancer cells. Cell Mol Life Sci.

[43] Huang S-B, Rivas P, Yang X, Lai Z, Chen Y, Schadler KL (2022). SIRT1 inhibition-induced senescence as a strategy to prevent prostate cancer progression. Mol Carcinog.

[44] Schmitt CA, Fridman JS, Yang M, Lee S, Baranov E, Hoffman RM (2002). A senescence program controlled by p53 and p16INK4a contributes to the outcome of cancer therapy. Cell.

[45] Schmitt CA (2007). Cellular senescence and cancer treatment. Biochim Biophys Acta.

[46] Roninson IB (2003). Tumor cell senescence in cancer treatment. Cancer Res.

[47] Xue W, Zender L, Miething C, Dickins RA, Hernando E, Krizhanovsky V (2007). Senescence and tumour clearance is triggered by p53 restoration in murine liver carcinomas. Nature.

[48] Petti C, Molla A, Vegetti C, Ferrone S, Anichini A, Sensi M (2006). Coexpression of NRASQ61R and BRAFV600E in human melanoma cells activates senescence and increases susceptibility to cell-mediated cytotoxicity. Cancer Res.

[49] Wang L, Lankhorst L, Bernards R (2022). Exploiting senescence for the treatment of cancer. Nat Rev Cancer.

[50] Schmitt CA, Wang B, Demaria M (2022). Senescence and cancer—role and therapeutic opportunities. Nat Rev Clin Oncol.

[51] Baskar R, Itahana K (2017). Radiation therapy and cancer control in developing countries: can we save more lives?. Int J Med Sci.

[52] Frame FM, Savoie H, Bryden F, Giuntini F, Mann VM, Simms MS (2016). Mechanisms of growth inhibition of primary prostate epithelial cells following gamma irradiation or photodynamic therapy include senescence, necrosis, and autophagy, but not apoptosis. Cancer Med.

[53] Baskar R, Dai J, Wenlong N, Yeo R, Yeoh K-W (2014). Biological response of cancer cells to radiation treatment. Front Mol Biosci.

[54] Lehmann BD, McCubrey JA, Jefferson HS, Paine MS, Chappell WH, Terrian DM (2007). A dominant role for p53-dependent cellular senescence in radiosensitization of human prostate cancer cells. Cell Cycle.

[55] Fang Y, DeMarco VG, Nicholl MB (2012). Resveratrol enhances radiation sensitivity in prostate cancer by inhibiting cell proliferation and promoting cell senescence and apoptosis. Cancer Sci.

[56] Bromfield G, Meng A, Warde P, Bristow RG (2003). Cell death in irradiated prostate epithelial cells: role of apoptotic and clonogenic cell kill. Prostate Cancer Prostatic Dis.

[57] Crosby M, Jacobberger J, Gupta D, Macklis R, Almasan A (2007). E2F4 regulates a stable G2 arrest response to genotoxic stress in prostate carcinoma. Oncogene.

[58] Lehmann BD, McCubrey JA, Terrian DM (2007). Radiosensitization of prostate cancer by priming the wild-type p53-dependent cellular senescence pathway. Cancer Biol Ther.

[59] Jang M, Cai L, Udeani GO, Slowing KV, Thomas CF, Beecher CW (1997). Cancer chemopreventive activity of resveratrol, a natural product derived from grapes. Science..

[60] Shishodia S, Aggarwal BB, Shishodia S, Aggarwal BB (2005). Resveratrol: a polyphenol for all seasons. Resveratrol in health and disease.

[61] Bhardwaj A, Sethi G, Vadhan-Raj S, Bueso-Ramos C, Takada Y, Gaur U (2007). Resveratrol inhibits proliferation, induces apoptosis, and overcomes chemoresistance through down-regulation of STAT3 and nuclear factor-κB–regulated antiapoptotic and cell survival gene products in human multiple myeloma cells. Blood.

[62] Chatterjee P, Choudhary GS, Sharma A, Singh K, Heston WD, Ciezki J (2013). PARP inhibition sensitizes to low dose-rate radiation TMPRSS2-ERG fusion gene-expressing and PTEN-deficient prostate cancer cells. PLoS ONE.

[63] de Bono J, Mateo J, Fizazi K, Saad F, Shore N, Sandhu S (2020). Olaparib for metastatic castration-resistant prostate cancer. N Engl J Med.

[64] Sokolova AO, Yu EY, Cheng HH (2020). Honing in on PARPi response in prostate cancer: from HR pathway to gene-by-gene granularity. Clin Cancer Res.

[65] Barreto-Andrade JC, Efimova EV, Mauceri HJ, Beckett MA, Sutton HG, Darga TE (2011). Response of human prostate cancer cells and tumors to combining PARP inhibition with ionizing radiation. Mol Cancer Ther.

[66] Sharma A, Almasan A (2021). Autophagy and PTEN in DNA damage-induced senescence. Adv Cancer Res.

[67] Ewald JA, Desotelle JA, Church DR, Yang B, Huang W, Laurila TA (2013). Androgen deprivation induces senescence characteristics in prostate cancer cells in vitro and in vivo. Prostate.

[68] Burton DG, Giribaldi MG, Munoz A, Halvorsen K, Patel A, Jorda M (2013). Androgen deprivation-induced senescence promotes outgrowth of androgen-refractory prostate cancer cells. PLoS ONE.

[69] Kawata H, Kamiakito T, Nakaya T, Komatsubara M, Komatsu K, Morita T (2017). Stimulation of cellular senescent processes, including secretory phenotypes and anti-oxidant responses, after androgen deprivation therapy in human prostate cancer. J Steroid Biochem Mol Biol.

[70] Westin P, Stattin P, Damber JE, Bergh A (1995). Castration therapy rapidly induces apoptosis in a minority and decreases cell proliferation in a majority of human prostatic tumors. Am J Pathol.

[71] Schiewer MJ, Augello MA, Knudsen KE (2012). The AR dependent cell cycle: mechanisms and cancer relevance. Mol Cell Endocrinol.

[72] Balk SP, Knudsen KE (2008). AR, the cell cycle, and prostate cancer. Nucl Recept Signal.

[73] Kawabata R, Oie S, Takahashi M, Kanayama H, Oka T, Itoh K (2011). Up-regulation of insulin-like growth factor-binding protein 3 by 5-fluorouracil (5-FU) leads to the potent anti-proliferative effect of androgen deprivation therapy combined with 5-FU in human prostate cancer cell lines. Int J Oncol.

[74] Pernicová Z, Slabáková E, Kharaishvili G, Bouchal J, Král M, Kunická Z (2011). Androgen depletion induces senescence in prostate cancer cells through down-regulation of Skp2. Neoplasia..

[75] Barakat DJ, Zhang J, Barberi T, Denmeade SR, Friedman AD, Paz-Priel I (2015). CCAAT/Enhancer binding protein β controls androgen-deprivation-induced senescence in prostate cancer cells. Oncogene.

[76] Orjalo AV, Bhaumik D, Gengler BK, Scott GK, Campisi J (2009). Cell surface-bound IL-1α is an upstream regulator of the senescence-associated IL-6/IL-8 cytokine network. Proc Natl Acad Sci USA.

[77] Sebastian T, Malik R, Thomas S, Sage J, Johnson PF (2005). C/EBPbeta cooperates with RB:E2F to implement Ras(V12)-induced cellular senescence. EMBO J.

[78] Karantanos T, Corn PG, Thompson TC (2013). Prostate cancer progression after androgen deprivation therapy: mechanisms of castrate resistance and novel therapeutic approaches. Oncogene.

[79] Kishi H, Igawa M, Kikuno N, Yoshino T, Urakami S, Shiina H (2004). Expression of the survivin gene in prostate cancer: correlation with clinicopathological characteristics, proliferative activity and apoptosis. J Urol.

[80] Montgomery RB, Mostaghel EA, Vessella R, Hess DL, Kalhorn TF, Higano CS (2008). Maintenance of intratumoral androgens in metastatic prostate cancer: a mechanism for castration-resistant tumor growth. Cancer Res.

[81] Carpenter VJ, Patel BB, Autorino R, Smith SC, Gewirtz DA, Saleh T (2020). Senescence and castration resistance in prostate cancer: a review of experimental evidence and clinical implications. Biochim Biophys Acta Rev Cancer.

[82] Wagner J, Damaschke N, Yang B, Truong M, Guenther C, McCormick J (2015). Overexpression of the novel senescence marker β-galactosidase (GLB1) in prostate cancer predicts reduced PSA recurrence. PLoS ONE.

[83] Wang L, de Oliveira RL, Wang C, Neto JMF, Mainardi S, Evers B (2017). High-throughput functional genetic and compound screens identify targets for senescence induction in cancer. Cell Rep.

[84] Schweizer MT, Antonarakis ES, Denmeade SR (2017). Bipolar androgen therapy: a paradoxical approach for the treatment of castration-resistant prostate cancer. Eur Urol..

[85] Denmeade SR, Isaacs JT (2010). Bipolar androgen therapy: the rationale for rapid cycling of supraphysiologic androgen/ablation in men with castration resistant prostate cancer. Prostate.

[86] Roediger J, Hessenkemper W, Bartsch S, Manvelyan M, Huettner SS, Liehr T (2014). Supraphysiological androgen levels induce cellular senescence in human prostate cancer cells through the Src-Akt pathway. Mol Cancer.

[87] Leone G, Buttigliero C, Pisano C, Di Stefano RF, Tabbò F, Turco F (2020). Bipolar androgen therapy in prostate cancer: current evidences and future perspectives. Crit Rev Oncol Hematol.

[88] Pungsrinont T, Sutter MF, Ertingshausen MC, Lakshmana G, Kokal M, Khan AS (2020). Senolytic compounds control a distinct fate of androgen receptor agonist-and antagonist-induced cellular senescent LNCaP prostate cancer cells. Cell Biosci.

[89] Bartsch S, Mirzakhani K, Neubert L, Stenzel A, Ehsani M, Esmaeili M (2021). Antithetic hTERT regulation by androgens in prostate cancer cells: hTERT inhibition is mediated by the ING1 and ING2 tumor suppressors. Cancers.

[90] Mirzakhani K, Kallenbach J, Rasa SMM, Ribaudo F, Ungelenk M, Ehsani M (2022). The androgen receptor—lncRNASAT1-AKT-p15 axis mediates androgen-induced cellular senescence in prostate cancer cells. Oncogene.

[91] Hessenkemper W, Roediger J, Bartsch S, Houtsmuller AB, van Royen ME, Petersen I (2014). A natural androgen receptor antagonist induces cellular senescence in prostate cancer cells. Mol Endocrinol.

[92] McCormick JR, Blute ML, Yang B, Damaschke N, Jarrard DF (2016). MP50-05 Synthetic lethal metabolic targeting of cellular senescence in prostate cancer with the repurposed drug metformin. J Urol.

[93] Kokal M, Mirzakhani K, Pungsrinont T, Baniahmad A (2020). Mechanisms of androgen receptor agonist-and antagonist-mediated cellular senescence in prostate cancer. Cancers.

[94] Gupta S, Pungsrinont T, Ženata O, Neubert L, Vrzal R, Baniahmad A (2020). Interleukin-23 represses the level of cell senescence induced by the androgen receptor antagonists enzalutamide and darolutamide in castration-resistant prostate cancer cells. Horm Cancer.

[95] Fousteris MA, Schubert U, Roell D, Roediger J, Bailis N, Nikolaropoulos SS (2010). 20-Aminosteroids as a novel class of selective and complete androgen receptor antagonists and inhibitors of prostate cancer cell growth. Bioorg Med Chem.

[96] Tran C, Ouk S, Clegg NJ, Chen Y, Watson PA, Arora V (2009). Development of a second-generation antiandrogen for treatment of advanced prostate cancer. Science.

[97] Rodriguez-Vida A, Galazi M, Rudman S, Chowdhury S, Sternberg CN (2015). Enzalutamide for the treatment of metastatic castration-resistant prostate cancer. Drug Des Dev Ther.

[98] Ghashghaei M, Muanza T, Paliouras M, Niazi T (2017). Effect of enzalutamide on sensitivity in prostate cancer cells to radiation by inhibition of DNA double strand break repair. Am Soc Clin Oncol.

[99] Ghashghaei M, Paliouras M, Heravi M, Bekerat H, Trifiro M, Niazi TM (2018). Enhanced radiosensitization of enzalutamide via schedule dependent administration to androgen-sensitive prostate cancer cells. Prostate.

[100] Fizazi K, Shore N, Tammela TL, Ulys A, Vjaters E, Polyakov S (2019). Darolutamide in nonmetastatic, castration-resistant prostate cancer. N Engl J Med.

[101] Ehsani M, Bartsch S, Rasa SMM, Dittmann J, Pungsrinont T, Neubert L (2022). The natural compound atraric acid suppresses androgen-regulated neo-angiogenesis of castration-resistant prostate cancer through angiopoietin 2. Oncogene.

[102] Roell D, Rösler TW, Hessenkemper W, Kraft F, Hauschild M, Bartsch S (2019). Halogen-substituted anthranilic acid derivatives provide a novel chemical platform for androgen receptor antagonists. J Steroid Biochem Mol Biol.

[103] Goktas S, Crawford ED (1999). Optimal hormonal therapy for advanced prostatic carcinoma. Semin Oncol.

[104] Petrylak DP (2006). The treatment of hormone-refractory prostate cancer: docetaxel and beyond. Rev Urol..

[105] Tannock IF, de Wit R, Berry WR, Horti J, Pluzanska A, Chi KN (2004). Docetaxel plus prednisone or mitoxantrone plus prednisone for advanced prostate cancer. N Engl J Med.

[106] Gilligan T, Kantoff PW (2002). Chemotherapy for prostate cancer. Urology..

[107] Yang X, Chen H, Xu D, Chen X, Li Y, Tian J (2022). Efficacy and safety of Androgen Deprivation Therapy (ADT) combined with modified docetaxel chemotherapy versus ADT combined with standard docetaxel chemotherapy in patients with metastatic castration-resistant prostate cancer: study protocol for a multicentre prospective randomized controlled trial. BMC Cancer.

[108] Sweeney CJ, Chen Y-H, Carducci M, Liu G, Jarrard DF, Eisenberger M (2015). Chemohormonal therapy in metastatic hormone-sensitive prostate cancer. N Engl J Med.

[109] Sun G, Chen X, Gong U, Chen Y, Li G, Wei F (2019). Androgen deprivation therapy with chemotherapy or abiraterone for patients with metastatic hormone-naive prostate cancer: a systematic review and meta-analysis. Future Oncol.

[110] Hickman JA (1992). Apoptosis induced by anticancer drugs. Cancer Metastasis Rev.

[111] Waldman T, Lengauer C, Kinzler KW, Vogelstein B (1996). Uncoupling of S phase and mitosis induced by anticancer agents in cells lacking p21. Nature.

[112] Bunz F, Dutriaux A, Lengauer C, Waldman T, Zhou S, Brown JP (1998). Requirement for p53 and p21 to sustain G2 arrest after DNA damage. Science.

[113] Kung AL, Zetterberg A, Sherwood SW, Schimke RT (1990). Cytotoxic effects of cell cycle phase specific agents: result of cell cycle perturbation. Cancer Res.

[114] Chang BD, Broude EV, Dokmanovic M, Zhu H, Ruth A, Xuan Y (1999). A senescence-like phenotype distinguishes tumor cells that undergo terminal proliferation arrest after exposure to anticancer agents. Cancer Res.

[115] Ewald JA, Peters N, Desotelle JA, Hoffmann FM, Jarrard DF (2009). A high-throughput method to identify novel senescence-inducing compounds. J Biomol Screen.

[116] Schwarze SR, Fu VX, Desotelle JA, Kenowski ML, Jarrard DF (2005). The identification of senescence-specific genes during the induction of senescence in prostate cancer cells. Neoplasia.

[117] Chang BD, Swift ME, Shen M, Fang J, Broude EV, Roninson IB (2002). Molecular determinants of terminal growth arrest induced in tumor cells by a chemotherapeutic agent. Proc Natl Acad Sci USA.

[118] Vergel M, Marin JJ, Estevez P, Carnero A (2010). Cellular senescence as a target in cancer control. J Aging Res.

[119] Minotti G, Menna P, Salvatorelli E, Cairo G, Gianni L (2004). Anthracyclines: molecular advances and pharmacologic developments in antitumor activity and cardiotoxicity. Pharmacol Rev.

[120] Chen W, Liu I, Tomiyasu H, Lee J, Cheng C, Liao AT (2019). Imatinib enhances the anti-tumour effect of doxorubicin in canine B-cell lymphoma cell line. Vet J.

[121] Ewald J, Desotelle J, Almassi N, Jarrard D (2008). Drug-induced senescence bystander proliferation in prostate cancer cells in vitro and in vivo. Br J Cancer.

[122] Suwiwat S, Ricciardelli C, Tammi R, Tammi M, Auvinen P, Kosma V-M (2004). Expression of extracellular matrix components versican, chondroitin sulfate, tenascin, and hyaluronan, and their association with disease outcome in node-negative breast cancer. Clin Cancer Res.

[123] Schwarze SR, Luo J, Isaacs WB, Jarrard DF (2005). Modulation of CXCL14 (BRAK) expression in prostate cancer. Prostate.

[124] Bender JF, Grillo-Lopez AJ, Posada JG (1983). Diaziquone (AZQ). Invest New Drugs.

[125] Ewald JA, Jarrard DF (2012). Decreased Skp2 expression is necessary but not sufficient for therapy-induced senescence in prostate cancer. Transl Oncol.

[126] Loda M (2000). p27KIP1: androgen regulation and prognostic significance in prostate cancer. Adv Clin Path.

[127] Seo SR, Chong SA, Lee SI, Sung JY, Ahn YS, Chung KC (2001). Zn2+-induced ERK activation mediated by reactive oxygen species causes cell death in differentiated PC12 cells. J Neurochem.

[128] Carraway RE, Dobner PR (2012). Zinc pyrithione induces ERK- and PKC-dependent necrosis distinct from TPEN-induced apoptosis in prostate cancer cells. Biochim Biophys Acta.

[129] Braig M, Lee S, Loddenkemper C, Rudolph C, Peters AH, Schlegelberger B (2005). Oncogene-induced senescence as an initial barrier in lymphoma development. Nature.

[130] Acosta JC, O'Loghlen A, Banito A, Guijarro MV, Augert A, Raguz S (2008). Chemokine signaling via the CXCR2 receptor reinforces senescence. Cell.

[131] Baker DJ, Wijshake T, Tchkonia T, LeBrasseur NK, Childs BG, Van De Sluis B (2011). Clearance of p16Ink4a-positive senescent cells delays ageing-associated disorders. Nature.

[132] Freund A, Patil CK, Campisi J (2011). p38MAPK is a novel DNA damage response-independent regulator of the senescence-associated secretory phenotype. EMBO J.

[133] Bavik C, Coleman I, Dean JP, Knudsen B, Plymate S, Nelson PS (2006). The gene expression program of prostate fibroblast senescence modulates neoplastic epithelial cell proliferation through paracrine mechanisms. Can Res.

[134] Krtolica A, Parrinello S, Lockett S, Desprez P-Y, Campisi J (2001). Senescent fibroblasts promote epithelial cell growth and tumorigenesis: a link between cancer and aging. Proc Natl Acad Sci USA.

[135] Kramer G, Marberger M (2006). Could inflammation be a key component in the progression of benign prostatic hyperplasia?. Curr Opin Urol.

[136] Coppé J-P, Kauser K, Campisi J, Beauséjour CM (2006). Secretion of vascular endothelial growth factor by primary human fibroblasts at senescence. J Biol Chem.

[137] Currid CA, O'Connor DP, Chang BD, Gebus C, Harris N, Dawson KA (2006). Proteomic analysis of factors released from p21-overexpressing tumour cells. Proteomics.

[138] Chang B-D, Watanabe K, Broude EV, Fang J, Poole JC, Kalinichenko TV (2000). Effects of p21Waf1/Cip1/Sdi1 on cellular gene expression: implications for carcinogenesis, senescence, and age-related diseases. Proc Natl Acad Sci USA.

[139] Khwaja F, Svoboda P, Reed M, Pohl J, Pyrzynska B, Van Meir E (2006). Proteomic identification of the wt-p53-regulated tumor cell secretome. Oncogene.

[140] Gilbert LA, Hemann MT (2010). DNA damage-mediated induction of a chemoresistant niche. Cell.

[141] Zhang B, Fu D, Xu Q, Cong X, Wu C, Zhong X (2018). The senescence-associated secretory phenotype is potentiated by feedforward regulatory mechanisms involving Zscan4 and TAK1. Nat Commun.

[142] Zhao B, Xu W, Rong B, Chen G, Ye X, Dai R (2018). H3K14me3 genomic distributions and its regulation by KDM4 family demethylases. Cell Res.

[143] Zhang B, Long Q, Wu S, Xu Q, Song S, Han L (2021). KDM4 orchestrates epigenomic remodeling of senescent cells and potentiates the senescence-associated secretory phenotype. Nat Aging.

[144] Lehmann BD, Paine MS, Brooks AM, McCubrey JA, Renegar RH, Wang R (2008). Senescence-associated exosome release from human prostate cancer cells. Can Res.

[145] Kowal J, Arras G, Colombo M, Jouve M, Morath JP, Primdal-Bengtson B (2016). Proteomic comparison defines novel markers to characterize heterogeneous populations of extracellular vesicle subtypes. Proc Natl Acad Sci.

[146] Zhang H, Freitas D, Kim HS, Fabijanic K, Li Z, Chen H (2018). Identification of distinct nanoparticles and subsets of extracellular vesicles by asymmetric flow field-flow fractionation. Nat Cell Biol.

[147] Zhang H, Li M, Zhang J, Shen Y, Gui Q (2021). Exosomal Circ-XIAP promotes docetaxel resistance in prostate cancer by regulating miR-1182/TPD52 axis. Drug Des Dev Ther.

[148] Takasugi M, Okada R, Takahashi A, Virya Chen D, Watanabe S, Hara E (2017). Small extracellular vesicles secreted from senescent cells promote cancer cell proliferation through EphA2. Nat Commun.

[149] Sancho-Albero M, Navascués N, Mendoza G, Sebastián V, Arruebo M, Martín-Duque P (2019). Exosome origin determines cell targeting and the transfer of therapeutic nanoparticles towards target cells. J Nanobiotechnology.

[150] Chowdhury SG, Ray R, Bhattacharya D, Karmakar P (2022). DNA damage induced cellular senescence and it’s PTEN-armed exosomes—the warriors against prostate carcinoma cells. Med Oncol.

[151] Wu M, Ouyang Y, Wang Z, Zhang R, Huang P-H, Chen C (2017). Isolation of exosomes from whole blood by integrating acoustics and microfluidics. Proc Natl Acad Sci USA.

[152] Takahashi K, Yan IK, Kogure T, Haga H, Patel T (2014). Extracellular vesicle-mediated transfer of long non-coding RNA ROR modulates chemosensitivity in human hepatocellular cancer. FEBS Open Bio.

[153] Lee S, Kim S, Chung H, Moon JH, Kang SJ, Park C-G (2020). Mesenchymal stem cell-derived exosomes suppress proliferation of T cells by inducing cell cycle arrest through p27kip1/Cdk2 signaling. Immunol Lett.

[154] Patel GK, Khan MA, Bhardwaj A, Srivastava SK, Zubair H, Patton MC (2017). Exosomes confer chemoresistance to pancreatic cancer cells by promoting ROS detoxification and miR-155-mediated suppression of key gemcitabine-metabolising enzyme, DCK. Br J Cancer.

[155] Zeng A, Yan W, Liu Y, Wang Z, Hu Q, Nie E (2017). Tumour exosomes from cells harbouring PTPRZ1–MET fusion contribute to a malignant phenotype and temozolomide chemoresistance in glioblastoma. Oncogene.

[156] Burdakov V, Kovalev R, Pantina R, Varfolomeeva EY, Makarov E, Filatov M (2018). Exosomes transfer p53 between cells and can suppress growth and proliferation of p53-negative cells. Cell Tiss Biol.

[157] Chowdhury R, Webber JP, Gurney M, Mason MD, Tabi Z, Clayton A (2015). Cancer exosomes trigger mesenchymal stem cell differentiation into pro-angiogenic and pro-invasive myofibroblasts. Oncotarget.

[158] Peak TC, Panigrahi GK, Praharaj PP, Su Y, Shi L, Chyr J (2020). Syntaxin 6-mediated exosome secretion regulates enzalutamide resistance in prostate cancer. Mol Carcinog.

[159] Ye Y, Li S-L, Ma Y-Y, Diao Y-J, Yang L, Su M-Q (2017). Exosomal miR-141-3p regulates osteoblast activity to promote the osteoblastic metastasis of prostate cancer. Oncotarget.

[160] Li SL, An N, Liu B, Wang SY, Wang JJ, Ye Y (2019). Exosomes from LNCaP cells promote osteoblast activity through miR-375 transfer. Oncol Lett.

[161] Hashimoto K, Ochi H, Sunamura S, Kosaka N, Mabuchi Y, Fukuda T (2018). Cancer-secreted hsa-miR-940 induces an osteoblastic phenotype in the bone metastatic microenvironment via targeting ARHGAP1 and FAM134A. Proc Natl Acad Sci USA.

[162] Wang X, Zhang H, Yang H, Bai M, Ning T, Deng T (2020). Exosome-delivered circRNA promotes glycolysis to induce chemoresistance through the miR-122-PKM2 axis in colorectal cancer. Mol Oncol.

[163] Del Re M, Biasco E, Crucitta S, Derosa L, Rofi E, Orlandini C (2017). The detection of androgen receptor splice variant 7 in plasma-derived exosomal RNA strongly predicts resistance to hormonal therapy in metastatic prostate cancer patients. Eur Urol.

[164] Takeda M, Mizokami A, Mamiya K, Li YQ, Zhang J, Keller ET (2007). The establishment of two paclitaxel-resistant prostate cancer cell lines and the mechanisms of paclitaxel resistance with two cell lines. Prostate.

[165] Kato T, Mizutani K, Kameyama K, Kawakami K, Fujita Y, Nakane K (2015). Serum exosomal P-glycoprotein is a potential marker to diagnose docetaxel resistance and select a taxoid for patients with prostate cancer. Urol Oncol.

[166] Kato T, Mizutani K, Kawakami K, Fujita Y, Ehara H, Ito M (2020). CD44v8-10 mRNA contained in serum exosomes as a diagnostic marker for docetaxel resistance in prostate cancer patients. Heliyon.

[167] Elgamal AA, Holmes EH, Su SL, Tino WT, Simmons SJ, Peterson M (2000). Prostate-specific membrane antigen (PSMA): current benefits and future value. Semin Surg Oncol.

[168] Mizutani K, Terazawa R, Kameyama K, Kato T, Horie K, Tsuchiya T (2014). Isolation of prostate cancer-related exosomes. Anticancer Res.

[169] Fuhrmann-Stroissnigg H, Niedernhofer LJ, Robbins PD (2018). Hsp90 inhibitors as senolytic drugs to extend healthy aging. Cell Cycle.

[170] Fuhrmann-Stroissnigg H, Ling YY, Zhao J, McGowan SJ, Zhu Y, Brooks RW (2017). Identification of HSP90 inhibitors as a novel class of senolytics. Nat Commun.

[171] Zhu Y, Doornebal EJ, Pirtskhalava T, Giorgadze N, Wentworth M, Fuhrmann-Stroissnigg H (2017). New agents that target senescent cells: the flavone, fisetin, and the BCL-XL inhibitors, A1331852 and A1155463. Aging (Albany NY).

[172] Huart C, Fransolet M, Demazy C, Le Calvé B, Lucas S, Michiels C (2022). Taking advantage of the senescence-promoting effect of olaparib after X-ray and proton irradiation using the senolytic drug, ABT-263. Cancers.

[173] Chang J, Wang Y, Shao L, Laberge R-M, Demaria M, Campisi J (2016). Clearance of senescent cells by ABT263 rejuvenates aged hematopoietic stem cells in mice. Nat Med.

[174] Wang H, Guo M, Wei H, Chen Y (2021). Targeting MCL-1 in cancer: current status and perspectives. J Hematol Oncol.

[175] Sancho M, Leiva D, Lucendo E, Orzáez M (2021). Understanding MCL1: from cellular function and regulation to pharmacological inhibition. FEBS J.

[176] Kotschy A, Szlavik Z, Murray J, Davidson J, Maragno AL, Toumelin-Braizat L (2016). The MCL1 inhibitor S63845 is tolerable and effective in diverse cancer models. Nature.

[177] Troiani M, Colucci M, D’Ambrosio M, Guccini I, Pasquini E, Varesi A (2022). Single-cell transcriptomics identifies Mcl-1 as a target for senolytic therapy in cancer. Nat Commun.

[178] Chaib S, Tchkonia T, Kirkland JL (2022). Cellular senescence and senolytics: the path to the clinic. Nat Med..

[179] Moiseeva O, Deschênes-Simard X, St-Germain E, Igelmann S, Huot G, Cadar AE (2013). Metformin inhibits the senescence-associated secretory phenotype by interfering with IKK/NF-κ B activation. Aging Cell.

[180] Laberge R-M, Sun Y, Orjalo AV, Patil CK, Freund A, Zhou L (2015). MTOR regulates the pro-tumorigenic senescence-associated secretory phenotype by promoting IL1A translation. Nat Cell Biol.

[181] Sun Y, Campisi J, Higano C, Beer TM, Porter P, Coleman I (2012). Treatment-induced damage to the tumor microenvironment promotes prostate cancer therapy resistance through WNT16B. Nat Med.

[182] Wang X, Ma L, Pei X, Wang H, Tang X, Pei J-F (2022). Comprehensive assessment of cellular senescence in the tumor microenvironment. Brief Bioinform.

[183] Feng D, Li D, Shi X, Xiong Q, Zhang F, Wei Q (2022). A gene prognostic index from cellular senescence predicting metastasis and radioresistance for prostate cancer. J Transl Med.

[184] Esmaeili M, Jennek S, Ludwig S, Klitzsch A, Kraft F, Melle C (2016). The tumor suppressor ING1b is a novel corepressor for the androgen receptor and induces cellular senescence in prostate cancer cells. J Mol Cell Biol.

[185] Guerrero J, Alfaro IE, Gómez F, Protter AA, Bernales S (2013). Enzalutamide, an androgen receptor signaling inhibitor, induces tumor regression in a mouse model of castration-resistant prostate cancer. Prostate.

